# The receptor tyrosine kinase AXL promotes migration and invasion in colorectal cancer

**DOI:** 10.1371/journal.pone.0179979

**Published:** 2017-07-20

**Authors:** Diana J. Uribe, Edward K. Mandell, Adam Watson, Jesse D. Martinez, Jonathan A. Leighton, Sourav Ghosh, Carla V. Rothlin

**Affiliations:** 1 Arizona Cancer Center, University of Arizona, Tucson, Arizona, United States of America; 2 Division of Gastroenterology, Mayo Clinic Arizona, Scottsdale, Arizona, United States of America; 3 Department of Neurology, Pharmacology and Yale Cancer Center, School of Medicine, Yale University, New Haven, Connecticut, United States of America; 4 Department of Immunobiology, Pharmacology and Yale Cancer Center, School of Medicine, Yale University, New Haven, Connecticut, United States of America; University of Navarra, SPAIN

## Abstract

The receptor tyrosine kinases (RTKs) *TYRO3*, *AXL* and *MERTK* (TAM) have well-described oncogenic functions in a number of cancers. Notwithstanding, TAM RTKs are also potent and indispensable inhibitors of inflammation. The combined deletion of *Axl* and *Mertk* in mice enhances chronic inflammation and autoimmunity, including increased inflammation in the gut and colitis-associated cancer. On the other hand, deletion of *Tyro3* increases the risk of allergic responses. Therefore, the indiscriminate inhibition of these TAM RTKs could result in undesirable immunological diseases. Here we show that *AXL*, but not *MERTK* or *TYRO3* expression is enhanced in late stage colorectal cancer (CRC) and *AXL* expression associates with a cell migration gene signature. Silencing *AXL* or the inhibition of AXL kinase activity significantly inhibits tumor cell migration and invasion. These results indicate that the selective inhibition of AXL alone might confer sufficient therapeutic benefit in CRC, while preserving at least some of the beneficial, anti-inflammatory effects of MERTK and TYRO3 RTKs.

## Introduction

RTKs have important roles in the development and growth of cancers [1, 2] and are ideal therapeutic targets. *TYRO3*, *AXL* and *MERTK* form a receptor tyrosine kinase RTK subfamily known as the TAM receptors [3, 4]. All three of the TAM receptors were independently cloned from cancer cells [5, 6]. These receptors have been associated with a number of human cancers including glioblastoma, melanoma, non-small cell lung cancer, breast cancer, hepatocellular carcinoma, ovarian cancer, pancreatic adenocarcinoma, head and neck cancer as well as in leukemias and lymphomas [5–8]. Furthermore, the mechanisms by which TAM RTKs promote tumors, such as cell migration/invasion, growth and survival, chemoresistance and/or angiogenesis signaling, have been described [5, 6] making this RTK family an attractive therapeutic target. Targeting the kinase activity of TAM RTKs through the use of small molecule inhibitors has demonstrated therapeutic efficacy in preclinical animal studies including in models of triple negative breast cancer, pancreatic cancer and non-small cell lung cancer. Additionally, clinical trials are underway [5–7, 9–13].

Colorectal cancer (CRC) is the fourth most common cancer and second leading cause of cancer death in USA [14]. In 2016, there were 134,490 estimated new cases of cancers of the colon and rectum in USA and 49,190 people died from this disease [14]. Molecular cancer therapies for CRC seek to inactivate oncogenes, including RTKs, involved in tumor growth, survival and/or metastasis. Expression of some of the TAM RTKs has been previously examined in CRC and gastric cancers [15–24], however the results from some of these studies have been ambiguous. A study investigating RTK expression in the progression of CRC identified AXL as one of eight tyrosine kinases detected in a peritoneal metastasis of a primary colon cancer [18]. However, there were no differences in *AXL* expression between matched control and tumor tissues in most of the samples tested, except for the case of peritoneal metastasis and one additional case of liver metastases [18]. Similarly, in an independent study, low levels of AXL and TYRO3 were detected in normal colon tissue with no changes in expression in polyps and metastasis [19]. In contrast, recent studies have reported high *AXL* expression in CRC and a correlation of *AXL* expression with poor survival in this disease [15, 17, 23].

In addition to their oncogenic function, the TAM RTKs are important anti-inflammatory mediators [25–28]. TAM RTKs, function as pleiotropic negative regulators of inflammation by inhibiting signaling pathways that drive the activation of dendritic cells and macrophages, and the production of pro-inflammatory cytokines [25, 29, 30]. This presents an intriguing, apparent paradox regarding TAM inhibitors in cancer, especially in CRC. Since chronic inflammation is closely linked to the development of colitis and colitis-associated cancer (CAC)–a subtype of CRC [31], the loss/inhibition of TAM RTK function can increase the risk of colitis and CAC. Consistent with this idea, the simultaneous genetic deletion of *Axl* and *Mertk* has been demonstrated to result in enhanced dextran sodium sulfate (DSS)-induced inflammation in the intestine and increased intestinal polyps after azoxymethane (AOM)-DSS treatment, in comparison to similarly treated wild-type mouse [26]. Moreover, the TAM ligand Gas6 has a similar tumor suppressive role. Loss of Gas6 results in increased CRC in AOM-DSS, as well as *Apc*^*min*^ mouse models [32].

TAM RTKs have a high degree of sequence identity [33] and the role of these individual RTKs in the negative regulation of inflammation was generally believed to be redundant. The immunological role of TAM RTKs was not revealed until the use of triple knockout approach where all three receptors were collectively ablated in mice [34]. In this study, the chronic inflammatory and autoimmune phenotypes were more significant in the triple knockout mice than in the combinatorial deletion of two TAM RTK members, which in turn were stronger than single knockouts. More recently, however, functional distinction and diversification between TAM RTKs have been discovered [28, 35]. Selective, rather than indiscriminate inhibition of all TAM RTKs, can preserve the anti-inflammatory role of this subfamily and may improve the therapeutic efficacy or safety window of TAM RTK inhibitors. Therefore, we sought to investigate if selective inhibition of a single TAM RTK can effectively inhibit the oncogenic function associated with this subfamily in CRC.

## Materials and methods

### Patients and tissue specimens

De-identified colorectal carcinoma specimens from 18 patients were obtained from a tissue repository at Arizona Cancer Center (AZCC) and retrospectively subjected to RT-qPCR analyses. Specimens for this existing tissue repository were collected with patients’ written consent for their use in research. After consultation with the University of Arizona Medical Center and the Arizona Cancer Center Review Board, this study was considered not to be human research as it involved existing, de-identified samples (exception #4) and IRB review was not required.

### Cell lines and drug treatments

RKO-AS45-1 and HCT-116 cell lines were purchased from American Type Culture Collection (ATCC, Manassas, VA). RKO-AS45-1 cells were grown in Dulbecco’s modified Eagle’s minimal essential medium (DMEM; Gibco BRL, Gaithersburg, MD, USA). HCT-116 cells were grown in RPMI 1640 (GIbco BRL) with L-Glutamine and 25 mM HEPES. Both cells were supplemented with 10% (v/v) fetal bovine serum (FBS; Gibco BRL), 100 U/ml penicillin and 100 mg/ml streptomycin (Gibco BRL) and kept at 37°C in a 5% CO_2_/95% air atmosphere. 5-FU and Camptothecin were purchased from Sigma and Cisplatin, Doxorubicin and R428 from Selleckchem. All drugs were solubilized in DMSO. Cells were treated with the drugs for 24 hr and cell viability was determined as described below.

### Extraction and purification of RNA

Approximately 250 mg of frozen tissue from each CRC patient sample was ground in RLT buffer (Qiagen) using a tissue homogenizer then purified following the RNeasy Mini Kit (Qiagen) protocol. For cancer cell lines, RLT buffer was directly added to plated cells and the lysate passed through a QIAShredder (Qiagen) before continuing with the RNeasy Mini Kit protocol.

### Reverse transcriptase-quantitative polymerase chain reaction (RT-qPCR)

After RNA purification, each sample was transcribed into cDNA using qScript cDNA SuperMix (Quanta BioSciences) or RT Superscript III (Invitrogen) or iScript cDNA Synthesis kit (BIO-RAD). The gene of interest was amplified using SYBR Green qPCR Master Mix (Applied Biosystems) and the 7500 Fast Real-Time PCR System (Applied Biosystems) or Stratagene Mx3000 System using KAPA SYBR Fast qPCR kit (KAPABIOSYSTEM). The reactions were normalized to housekeeping genes and the specificity of the amplified products was verified by dissociation curves. The primers sequences are described in S1 Table.

### RNA interference

siRNA targeting *AXL* were purchased from Sigma (sense: 5’-GUCAUCUUACC UUUCAUGAtt-3’ and antisense: 5’-UCAUGAAAGGUAAGAUGACtt-3’) and Ambion (sense: 5’-GGAACUGCAUGCUGAAUGAtt-3’ and antisense: 5’-UCAUUCAGCAUGCAGUUCCtg-3’). A scrambled siRNA sequence from the Ambion *AXL* siRNA was generated using InvivoGen’s siRNA Wizard online tool and the sequence ordered through Integrated DNA Technologies (IDT). Briefly, cells were transfected with 6, 12 or 25 nM of each siRNA using Lipofectamine RNAiMAX (Invitrogen), following the manufacturer’s recommended protocol. Cells were harvested or assayed after 48 hr of transfection.

### Cell proliferation assay

Cells were transfected and reseeded, 24 hr later, in a 96-well plate at a concentration of 1x10^4^ cells/mL and allowed to adhere to the plate before serum starving for 16 hr. Cell proliferation was determined using EdU incorporation via the Click-iT^®^ EdU Microplate Assay (Invitrogen) following the manufacturer’s recommended protocol. Fresh media containing 10% (v/v) FBS and 10 μM of EdU was added to the cells and incubated for 6 hr before continuing the EdU assay. All experiments were done in triplicate.

### Flow cytometry

Cells were seeded in a 10 cm plate and transfected with siRNA the following day. 48 hr after transfection, cells were harvested, washed twice in 1X PBS and slowly resuspended in cold 70% ethanol and left at -20°C overnight. The pelleted cells were washed, resuspended in PBS containing 500 ng/mL RNAse A and 40 ng/mL propidium iodide and incubating for 30 min at 37°C. Flow cytometry analysis was done within an hour after incubation. For apoptosis assay, ApoDETECT^™^ (Invitrogen), transfected cells were harvested, washed with PBS and resuspended in 1X binding buffer (10 mM Hepes/NaOH, pH 7.4, 140 mM NaCl, 2.5 mM CaCl2). 10 μL of Annexin V-FITC was added to 190 μL of cell suspension and incubated for 10 min at room temperature. The Annexin V-FITC was washed out using 1X binding buffer and resuspended in 190 μL of 1X binding buffer before adding 10 μL of 20 μg/mL propidium iodide. The stained cells were analyzed immediately after.

### Cell viability assay

Cells were seeded in a 96-well plate at a concentration of 1x10^4^ cells/mL and treated 16 hr after seeding. Cell viability was determined using the AlamarBlue^®^ (Invitrogen), 24 hr after treatment. Fluorescence of the AlamarBlue^®^ reagent was read using a peak excitation at 570 nm and peak emission at 585 nm using a Tecan Infinite M1000 scanner (Tecan Group Ltd).

### Transwell migration and invasion assay

For the migration assay, 1x10^5^ cells were seeded on a 24-well transwell with a 8-μm pore size membrane with no fetal bovine serum in the media. For the invasion assay, the day before the assay 200 μL of 1:10 (v/v) diluted matrigel in no-serum media was added on a 24-well transwell and allowed to solidify at 37°C overnight. The bottom of each 24-well contained complete media with 20% fetal bovine serum and the cells were allowed to migrate through the membrane overnight. The following day, the cells on the transwells were washed twice with PBS, fixed using 4% paraformaldehyde in 1X PBS, permeabilized using absolute methanol and stained with 0.05% crystal violet in 25% methanol. The non-migrating or invading cells on the inside of the transwells were taken off using a cotton swab. The migrated or invaded cells at the bottom of each transwell membrane were imaged using an inverted microscope Axiovert 40 CFL (Zeiss) and the image software Axiovision Rel 4.8 (Zeiss). Quantitative analysis of the migrated or invaded cells was done using Image J (National Institutes of Health, Bethesda, MD) and the plugin Color Threshold.

### Scratch assay

2x10^5^ cells were seeded in a 6-well plate 24 hr prior to transfecting with siRNA. The cells were then transfected using Lipofectamine RNAiMAX (Invitrogen) as described above. The next day after the transfection, the cell monolayer in each well was scratched using a P200 pipette tip, the displaced cells were washed off twice with 1x PBS and replenished with complete media. The cells were incubated at 37°C and images were taken at 0, 24 and 48 hr after the scratch using an inverted microscope Axiovert 40 CFL (Zeiss) and the image software Axiovision Rel 4.8 (Zeiss). Quantitative analysis of the cells migrating into the scratch was done using Image J software.

### Bioinformatics and statistical analysis

Level 3 TCGA COAD and READ gene expression and clinical data was acquired from the Cancer Genome Browser. Excel was used to view, cross-reference, bin and analyze data. Pearson correlation coefficients were calculated using the CORREL function. Expression data is expressed as log 2 RSEM. Gene ontology was determined using DAVID. Statistical analyses were performed using Prism7 software (GraphPad Inc.). Differences between the means of experimental groups were analyzed by one-way or Two-way ANOVA or by t-test as appropriate. For ANOVA, Bonferroni multiple comparison test was used for comparisons. P values ≤ 0.05 were considered significant. Pearson correlation coefficients where calculated to measure the linear dependency between the expression of genes tested. The magnitude of the effect size was calculated using the Cohen’s *d* test. Additional database searches were performed using the Oncomine^™^ webtool.

## Results and discussion

### *AXL*, but not *MERTK* or *TYRO3*, expression correlates with CRC progression

**Fig 1:**
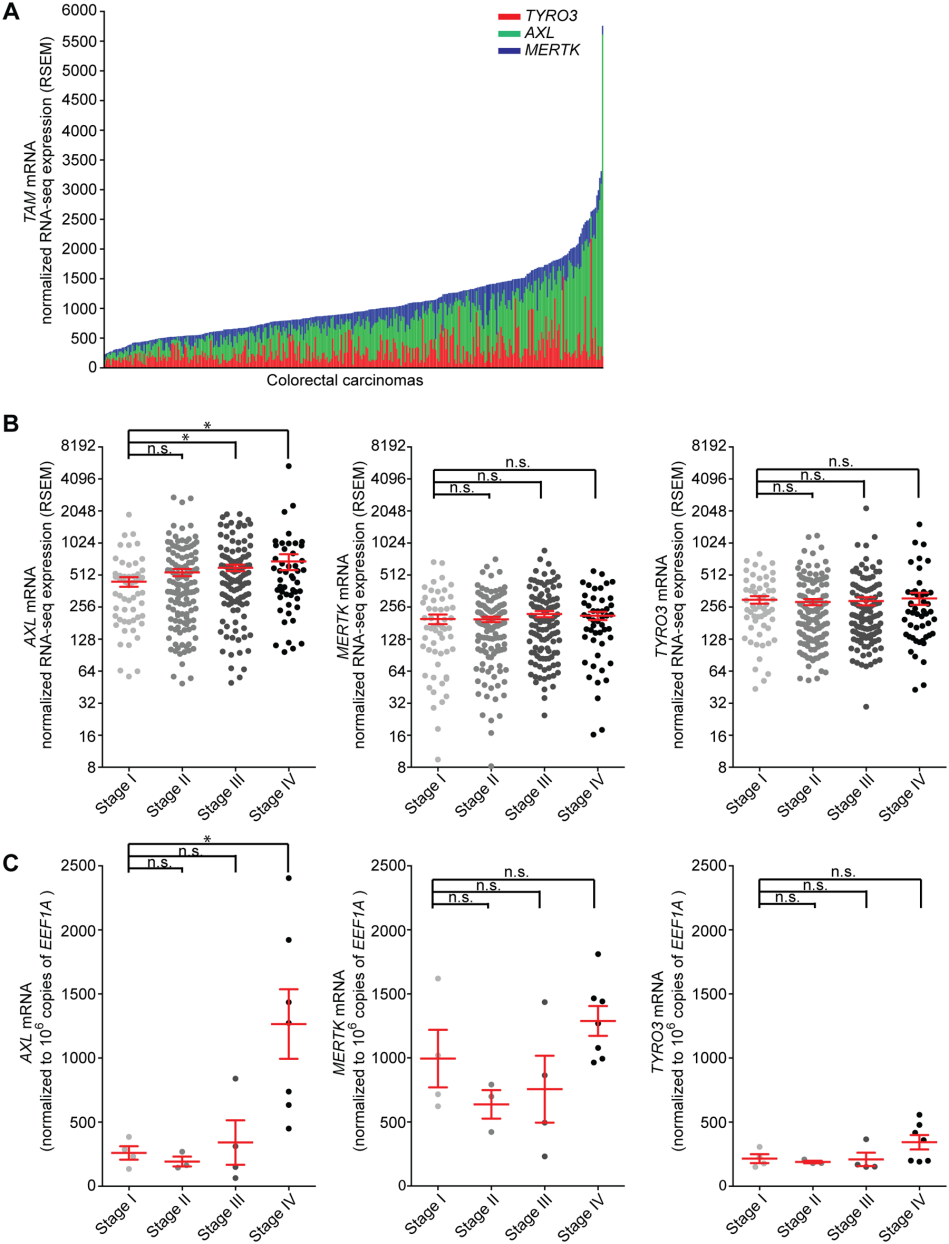
Increased expression of *AXL*, but not *MERTK* or *TYRO3*, in late state colorectal cancer. *(A)* Colorectal cancers stratified based on total expression of TAM RTKs from TCGA Illumina Hi-Seq RNA-seq data. Abundance of *TYRO3*, *AXL* and *MERTK* in each tumor (an individual bar) is represented by red, green and blue segments as indicated. *(B)* Abundance of *AXL*, *MERTK* and *TYRO3* in colon and rectal adenocarcinomas at the indicated stages based on RNA-seq data from TCGA data sets. Cohen’s *d* for change in *AXL* expression between Stage I versus Stage III colorectal cancer is 0.52 (moderate effect size) and between Stage I and Stage IV is 0.54 (moderate effect size). *(C)* Expression of *AXL*, *MERTK* and *TYRO3* in colorectal adenocarcinomas at the indicated stages, as detected by RT-qPCR. Data are presented as individual samples, mean ± SEM per tumor stage. * *p*<0.05; n.s., non significant.

We surveyed The Cancer Genome Atlas (TCGA) Illumina HiSeq 2000 datasets to assess the expression of all three TAM RTKs in colon adenocarcinomas (COAD) and rectal adenocarcinomas (READ) [36, 37]. Transcript quantitation was performed using RNA-Seq by Expectation Maximization (RSEM) [38]. The expression of all three TAM RTKs mRNAs was detected in CRC tissues, although the abundance varied between cases (Fig 1A). Next, we examined if the levels of TAM RTK expression changed with CRC stages. Our interrogation revealed that *AXL* expression in Stage III and IV colorectal adenocarcinomas was significantly enhanced in comparison to that in Stage I (Fig 1B). In contrast, no enrichment of *MERTK* or *TYRO3* was observed (Fig 1B). Analyses of additional colorectal datasets available through Oncomine^™^ similarly indicated a positive correlation of *AXL* expression with stage (S1 Fig). Subsequently, we obtained de-identified tissue from a total of 18 colorectal adenocarcinoma cases spanning Stage 0/I, Stage II, Stage III and Stage IV and examined TAM RTK expression by RT-qPCR using isoform specific primers and RT-qPCR. While a statistically significant increase in *AXL* expression correlated with Stage Stage IV CRC versus Stage 0/I (Fig 1C), *MERTK* or *TYRO3* expression did not show any change in abundance in Stage I-IV CRC (Fig 1C). Taken together our results indicate that although all three TAM RTKs were detected in CRC, only *AXL* expression positively correlated with disease progression. This raises the possibility that *AXL*, but not *MERTK* or *TYRO3*, may play a more active role in CRC progression and/or metastasis.

### *AXL*, but not *MERTK* or *TYRO3*, associates with a cell migration gene signature

**Fig 2:**
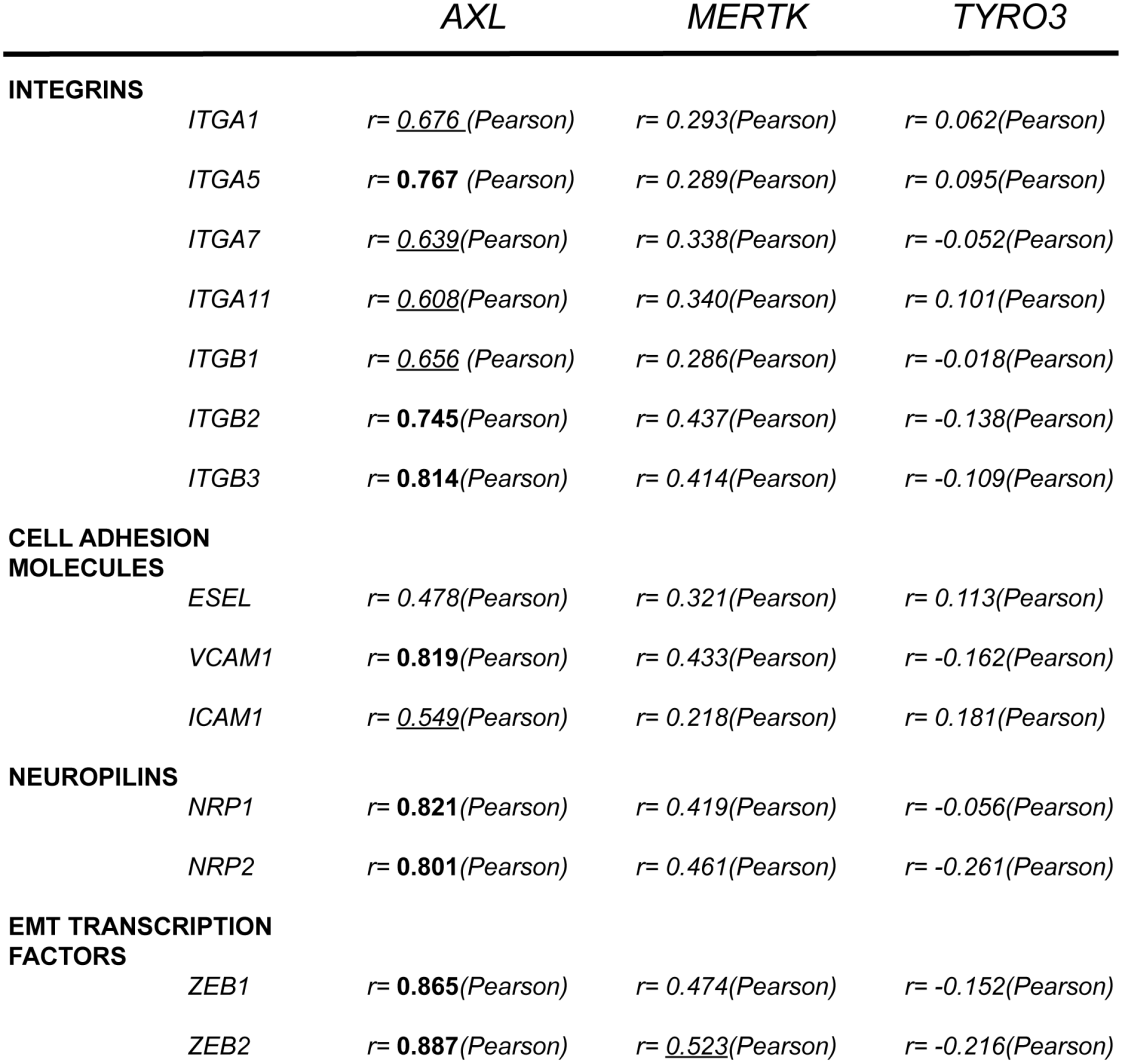
Expression of *AXL* correlates with the expression of cell migration-associated genes. Correlation between the expression of selected cell migration-associated genes, Integrins alpha 1, 5, 7 and 11, Integrins beta 1, 2 and 3, E-selectin, VCAM1, ICAM1, neuropilin 1 and 2 and ZEB1 and 2, and *AXL*, *MERTK* and *TYRO3* in TCGA. Strong Pearson correlation values > 0.7 are indicated in bold, while moderate Pearson correlation values (>0.5—<0.69) are underlined and in italics.

To identify a functional role of the TAM RTKs in CRC, initially we correlated the abundance of *TYRO3*, *AXL* and *MERTK* with the expression of other genes in the TCGA COAD and READ datasets. *AXL* expression strongly correlated with the expression levels of a large number of genes. Using a correlation cutoff of 0.5, 2120 genes positively and 13 negatively correlate with *AXL* (S2 Fig). In contrast, only 43 genes positively and 1 negatively correlated with *MERTK* and only 1 positively correlated with *TYRO3* by the same criteria (S2 Fig). Using DAVID [39, 40] to analyze functional annotation associated with gene groups that positively associated with *AXL*, we observed that 7 of the top 15 GO categories corresponded to cell adhesion, extracellular matrix organization and cell migration (S2 Table). In contrast, *MERTK*, which as discussed above positively correlated with only 43 genes, corresponded with protein localization and wound healing but with much lower P values (S3 Table). Subsequently, we examined the correlation of individual cell migration-associated genes such as integrins (*ITGA* and *ITGB*), cell adhesion molecules (E-selectin, *VCAM1* and *ICAM1*), neuropilins (*NRP1* and *NRP2*) and epithelial-to-mesenchymal transition transcription factors (*ZEB1* and *ZEB2*), with *AXL*, *MERTK* and *TYRO3* (Fig 2 and S3 Fig). Some of these genes, such as *ITGA5*, E-selectin (*SELE*), *VCAM1*, *ICAM1*, *NRP1* and *NRP2* have previously been implicated in CRC progression [41–45]. These genes associated strongly with *AXL*. Positive correlation with *MERTK* was less and with *TYRO3* even lesser or non-significant. These results suggest that *AXL*, but not *MERTK* or *TYRO3*, is part of the invasion signature in CRC. Although the TAM RTKs have been implicated in cell proliferation, growth and survival in some cancers, our analyses of COAD and READ data failed to uncover correlations with these GO categories. Our results are consistent with the report by Kimani *et al*. wherein AXL signaling, but not TYRO3 or MERTK signaling, was associated with migration and invasion signature in a series of unbiased screens [46].

### *AXL* silencing or the inhibition of AXL kinase activity is sufficient to reduce migration and invasion of CRC cells

To experimentally examine the molecular function of *AXL* in CRC *in vitro*, we tested a panel of human CRC cell lines for TAM RTK expression (S4 Fig). While RKO-AS45-1 had high levels of *AXL* expression, HCT116 showed lower levels of *AXL* expression. Caco2 did not have detectable expression of *AXL*; these cells only expressed *MERTK* and *TYRO3*. SW480 expressed *AXL* but both *MERTK* and *TYRO3* expression was undetectable in these cells. Since, AXL expression is not homogenous in CRC, we chose to examine the function of this gene in both RKO-AS45-1 (high expression) and HCT 116 (low expression). Both these cell lines also expressed *TYRO3* and low levels of *MERTK* consistent with our observations in human CRC. Next, we silenced *AXL* expression in RKO-AS45-1 and HCT116 using two independent siRNAs used at different concentrations (S4 Fig). We subsequently tested the effects of *AXL* silencing in cell migration and invasion in the CRC cell lines using siRNA #2.

**Fig 3:**
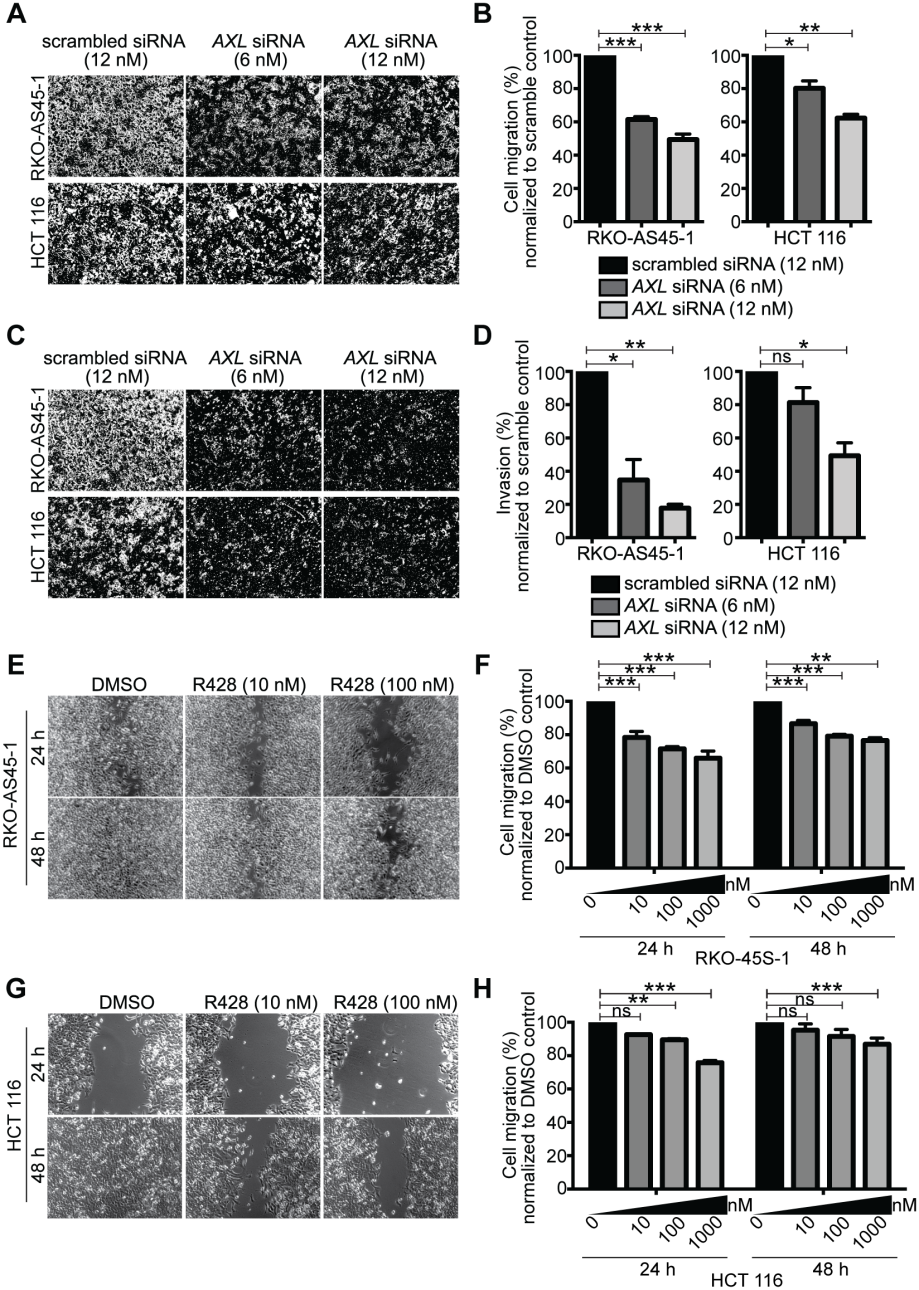
Silencing or inhibition of AXL kinase activity inhibits migration and invasion of human colon cancer cells. *(A)* Representative images and *(B)* relative cell migration in a Boyden chamber assay of RKO-AS45-1 and HCT116 cells transfected with scrambled or *AXL* siRNAs. *(C)* Representative images and *(D)* relative cell invasion in a Matrigel-coated Boyden chamber assay of RKO-AS45-1 and HCT116 cells transfected with scrambled or *AXL* siRNAs. *(E)* Representative images and *(F)* relative cell migration in a scratch wound healing assay of RKO-AS45-1 treated with the indicated concentrations of the small molecule AXL inhibitor, R428 after 24 or 48h. *(G)* Representative images and *(H)* relative cell migration in a scratch wound healing assay of HCT116 cultured in the presence of the indicated concentrations of the small molecule AXL inhibitor, R428 after 24 or 48h. Data is presented as representative samples or as mean ± SEM of 3 independent experiments, * *p*<0.05; ** *p*<0.01; *** *p*<0.001; n.s., non significant.

We tested if AXL is functionally involved in CRC cell migration and/or invasion. In a Boyden chamber assay, *AXL* silencing with either the 6 nM or 12 nM siRNA significantly reduced the chemotactic migration of RKO-AS45-1 (Fig 3A and 3B). HCT116 migration was also significantly inhibited at both the low and high concentration of *AXL* siRNA treatment (Fig 3A and 3B). Additionally, we tested CRC cell invasion using Matrigel-coated Boyden chamber assay. *AXL* siRNA treatment significantly inhibited invasion of RKO-AS45-1 through Matrigel (Fig 3C and 3D). A similar reduction in invasion was detected with HCT116 following *AXL* silencing, albeit only at the higher concentration of *AXL* siRNA treatment (Fig 3C and 3D).

To validate these results, we used a small molecule AXL inhibitor, R428, and examined the role of AXL kinase activity in regulating CRC cell migration. R428 has been reported to be selective for AXL as it has a 16-fold higher IC_50_ for MERTK and 14-fold higher IC_50_ for TYRO3 in biochemical assays [47]. Similarly, R428 has a 50-fold higher IC_50_ for MERTK and >100 fold higher IC_50_ for TYRO3 in cell-based assays [47]. In a scratch wound-healing assay, R428 treatment significantly reduced RKO-AS45-1 migration at 10 nM, 100 nM or 1000 nM concentrations at 24 and 48 hr (Fig 3E and 3F). In HCT116, migration was significantly inhibited by 100 nM and 1000 nM of R428 at 24 hr and by 1000 nM R428 at 48 hr (Fig 3G and 3H). Taken together, our results demonstrate that the silencing of *AXL* or the inhibition of AXL kinase activity with a selective inhibitor resulted in impairment of tumor cell migration, suggesting that AXL functions in CRC cell migration and metastasis. Importantly, the selective inhibition of *AXL* alone significantly reduced CRC cell migration and invasion. TAM receptors have been previously implicated in the regulation of actin cytoskeleton, cell elongation and epithelial-mesenchymal transition (EMT)–processes that influence metastatic potential. Most cancer-associated deaths occur due to metastasis. Therefore, inhibiting metastasis is expected to improve survival outcome in CRC. These results suggest that selective inhibition of *AXL* may reduce colon cancer progression.

### Selective targeting of *AXL* does not decrease proliferation/survival or increase apoptosis/chemo-sensitivity in CRC cells

**Fig 4:**
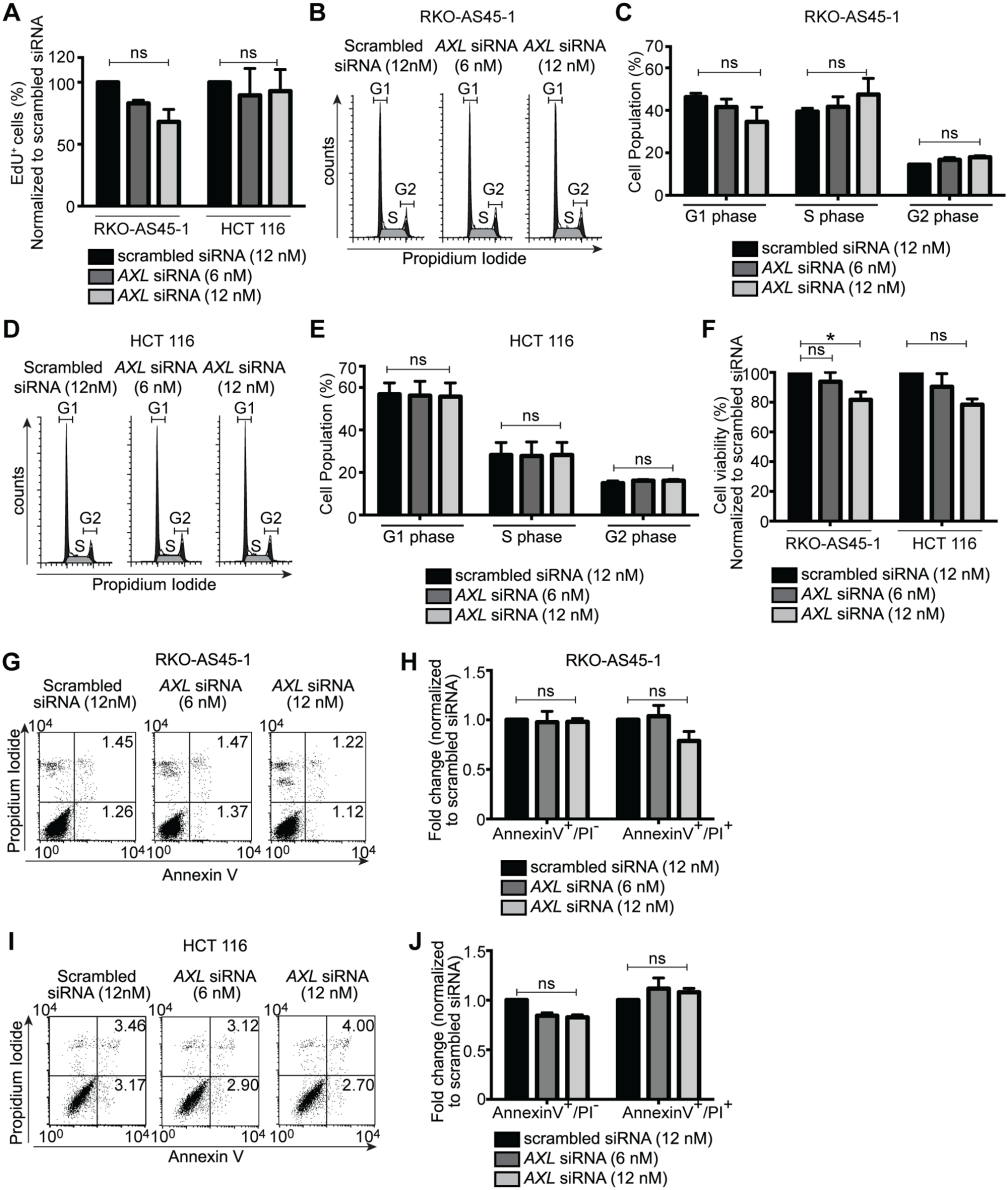
Silencing AXL in human colon cancer cells does not decrease proliferation. *(A)* Percentage of EdU incorporation normalized to scrambled siRNA control, in the indicated cell types transfected with scrambled or *AXL* siRNAs. *(B)* Representative FACS cell cycle analysis and *(C)* proportion of control siRNA or *AXL* siRNA-treated RKO-AS45-1 cells in G1, S and G2/M. *(D)* Representative FACS cell cycle analysis and *(E)* percentage of HCT116 cells at the indicated stages of the cell cycle after scrambled or *AXL* siRNAs transfection. *(F)* Percentage of viable RKO-AS45-1 and HCT116 after scrambled or *AXL* siRNAs transfection, as measured by Alamar Blue cell viability assay. *(G)* Representative FACS plots and *(H)* fold change in Annexin V^+^/Propidium Iodide (PI)^-^ and Annexin V^+^/(PI)^+^ RKO-AS45-1 cells after scrambled or *AXL* siRNAs transfection. *(I)* Representative FACS plots and *(J)* fold change in Annexin V^+^/Propidium Iodide (PI)^-^ and Annexin V^+^/(PI)^+^ HCT116 cells after scrambled or *AXL* siRNAs transfection. Data is presented as representative samples or as mean ± SEM of 3 independent experiments, * *p*<0.05; n.s., non significant.

Albeit that no significant correlation was observed between TAM RTK and cell proliferation/growth or survival in COAD and READ datasets, we experimentally tested if AXL regulates these aspects of cancer cell functions. *In vitro* proliferation assay demonstrated that EdU incorporation in RKO-AS45-1 and HCT116 was unchanged following *AXL* silencing using a lower and a higher concentration of *AXL* siRNA (Fig 4A). We validated these observations by propidium iodide labeling and FACS analyses of cell cycle (Fig 4B and 4D). Quantitation of these data indicated that there were no changes in the cell cycle characteristics of RKO-AS45-1 and HCT116 following *AXL* silencing (Fig 4C and 4E).

*AXL* has been demonstrated to regulate cell survival in a number of cancers by stimulating Akt and ERK1/2 anti-apoptotic pathways [6]. Therefore, we tested whether or not *AXL* silencing can affect cell survival in RKO-AS45-1 and HCT116. Alamar Blue cell viability assay indicated no significant difference in RKO-AS45-1 and HCT116 cell viability following *AXL* silencing, except for 12 nM siRNA in RKO-AS45-1 (Fig 4F). Similarly, propidium iodide and Annexin V staining to detect apoptotic cells failed to show statistically significant differences in RKO-AS45-1 and HCT116 after *AXL* silencing (Fig 4G and 4H).

We also investigated if *AXL* silencing enhanced chemosensitivity to agents that are used to treat CRC, including 5-Fluorouracil, Cisplatin, Camptothecin and Doxorubicin. LD_50_ of the reagents for wild type RKO-AS45-1 and HCT116 were individually determined and this concentration was used to treat *AXL*-silenced cells. Cell viability was monitored using Alamar Blue assay. RKO-AS45-1 showed a degree of increased sensitivity to cisplatin at both the low and high concentration of siRNA, while increased sensitivity of RKO-AS45-1 to doxorubicin was seen only at the high concentration of *AXL* siRNA (S5 Fig). HCT116 showed increased sensitivity to cisplatin, albeit only at the high *AXL* siRNA concentration (S5 Fig). Other than the exceptions noted above, the chemotherapeutic agents did not significantly alter RKO-AS45-1 and HCT116 cell viability following *AXL* silencing (S5 Fig). Collectively, our data indicates that the selective targeting of *AXL* neither reduced proliferation/survival, nor increased apoptosis/chemo-sensitivity at substantial levels, in the CRC cell lines tested.

## Conclusions

Our study demonstrates a functional distinction between members of the TAM RTKs in CRC. While all three TAM RTKs are expressed in CRC cells, AXL correlates with migration/invasion gene expression and the inhibition of *AXL* alone is sufficient to reduce tumor cell migration/invasion. In contrast, silencing *AXL* failed to inhibit proliferation and survival. These results are similar to those recently described by Kimani *et al*. using chimeric receptors indicating that *AXL* functions in migration/invasion while the TAM subfamily may redundantly function in cell proliferation and survival [46]. If the desirable anti-inflammatory effect of TAM RTKs can be segregated from the non-desirable oncogenic effects, the selective inhibition of a TAM subfamily member may largely preserve the anti-inflammatory function in immune cells of the intestinal mucosa. *Axl*^-/-^*Mertk*^-/-^ mice are significantly more susceptible to induced colitis and colitis-associated cancer in comparison to wild-type mouse [26], albeit that the effect of the individual genetic ablation of *Axl* or *Mertk* on intestinal inflammation in mouse models remains to be tested. Enhanced systemic inflammation is typically a more profound feature of *Axl*^-/-^*Mertk*^-/-^ double knockout mice, than *Axl*^-/-^ or *Mertk*^-/-^ single knockouts. This suggests some degree of additive or synergistic function of these RTKs in the regulation of the inflammatory response. Assuming that the expression and function of the TAM RTK isoforms in the human colon is evolutionarily conserved, a similar additivity or cooperativity of AXL and MERTK in regulating inflammation may be predicted. It is also possible that MERTK is the principal anti-inflammatory TAM RTK in human colon. Interestingly, levels of PROS1, which binds TYRO3 and MERTK with high affinity but not to AXL, are reduced in IBD patients [48]. Therefore, targeting AXL alone may reduce the risk of immune-related adverse events. Interestingly, a recent study found that TYRO3 amounts positively correlated with colorectal cancer progression [24]. The authors also observed that silencing *TYRO3* in HCT116 and HT29 colon cancer cell lines reduced cell migration and invasion [24]. While the difference between our studies and that of Chien *et al*. in *TYRO3* expression might reflect population-level differences, both studies suggest that inhibition of a single member of the TAM family may confer sufficient anti-cancer benefit. We have recently described *TYRO3* as an essential negative regulator of Type 2 immunity [28]. Loss of *Tyro3* exacerbated allergic asthma symptoms in a mouse model of house dust mite-induced airways inflammation. Selectively targeting AXL may also spare the desirable role of TYRO3 in limiting the immune response to allergens. TAM RTKs are known to facilitate the development of metastasis in a number of different cancers [5, 6, 49]. While our study is limited to understanding the functional specificity and diversity of TAM RTK in CRC, this paradigm of dissecting TAM RTK function can be important in the context of additional cancer types.

Unique features of the kinase active site of the TAM subfamily relative to other RTKs enable specific targeting of this subfamily [7]. Therefore, several small molecule inhibitors that target TAM RTKs have been designed [5, 7, 10]. While some inhibitors, for example R428, have been demonstrated to have *in vitro* and in cell selectivity profile favoring AXL inhibition over MERTK or TYRO3, the IC_50_ of other small molecule inhibitors are similar for all TAM RTKs [5, 47]. An additional approach of interfering with TAM RTK function is centered on neutralizing TAM ligands. Such approaches involve the use of TAM RTKs “decoy receptors” [50–52]. These approaches can also show some RTK selectivity. For example, soluble MERTK, AXL or TYRO3 ectodomains can neutralize the function of GAS6, an agonist for all TAM RTKs. Soluble MERTK or TYRO3 ectodomains will also neutralize PROS1, an agonist for MERTK and TYRO3. In contrast, soluble AXL ectodomain is expected to sequester GAS6, but spare PROS1 signaling through MERTK and TYRO3. In conclusion, uncoupling the specificity and functional diversity of the members of the TAM RTK subfamily can greatly benefit the understanding the biology of these RTKs and guide the rational use of anti-TAM RTK therapeutics.

## Supporting information

S1 Table List of primers. (PDF)

S2 Table GO and KEGG pathway categories associated with *AXL* expression in TCGA COAD and READ datasets. (PDF)

S3 Table GO categories associated with *MERTK* expression in TCGA COAD and READ datasets. (PDF)

S1 Fig Expression of *AXL* in the indicated colorectal cancer studies using the Oncomine^™^ webtool. (PDF)

S2 Fig Correlation between *TYRO3*, *AXL* and *MERTK* with the expression of other genes in the TCGA COAD and READ datasets. (XLSX)

S3 Fig Expression of *AXL* correlates with the expression of cell migration-associated genes. Correlation between the expression of selected cell migration-associated genes, Integrins alpha 1, 5, 7 and 11, Integrins beta 1, 2 and 3, E-selectin, VCAM1, ICAM1, Neuropilin 1 and 2 and ZEB1 and 2, and AXL, MERTK and TYRO3 in TCGA. mRNA amount is expressed as log2 RSEM.
(PDF)

S4 Fig TAM RTK expression in colon cancer cell lines and silencing. (A) expression of AXL, MERTK and TYRO3 in the indicated colon cancer cell lines, as detected by RT-qPCR. (B) representative western blot and (C) quantified AXL:ACTIN signal ratios in colon cancer cell lines. (D) representative western blot showing the efficiency of siRNA-mediated knock down of AXL in RKO-AS45-1 and HCT116 cells. Cells were transfected with scrambled or AXL siRNAs, cell lysates were collected after 48h, resolved by SDS/PAGE and immunoblotted with antibodies against AXL and ACTIN. Data are presented as individual samples or mean ± SEM of 3 independent samples.
(PDF)

S5 Fig Silencing AXL in human colon cancer cells does not substantially affect survival or chemo-sensitivity. Relative change in cell viability of RKO-AS45-1 and HCT116 transfected with scrambled or AXL siRNAs and treated with LD50 concentrations of the indicated chemotherapeutic agents for 24 hours. Data is presented as representative samples or as mean ± SEM of 3 independent experiments, * *p*<0.05; ** *p* <0.01; n.s., non significant.
(PDF)

## Abbreviations

TAM: TYRO3, AXL and MERTK
