# Supplementary material for: The receptor tyrosine kinase AXL promotes migration and invasion in colorectal cancer
Source: PLoS One. 2017 Jul 20;12(7):e0179979. doi: 10.1371/journal.pone.0179979 (PMC5519024; doi:10.1371/journal.pone.0179979)
Supplement: S1 Table — (PDF) [file pone.0179979.s001.pdf]

List of Primers

AXL forward 5’-CCAGGACACCCCAGAGGTGCTAAT-3’

AXL reverse 5’-TGGTGGACTGGCTGTGCTTGC-3’

MERTK forward 5’-GCCGTCACCAGAGGGGGAGT-3’

MERTK reverse 5’-TCTACCCAACCGTGTGCAGGGA-3’

TYRO3 forward 5’-GCTAACGGCCCTGGTGACGG-3’

TYRO3 reverse 5’-AGGCTTGCCCAAACCGCGTC-3’

EEF1A forward 5’-TCGGGCAAGTCCACCACTAC-3’,

EEF1A reverse 5’-CCAAGACCCAGGCATACTTGA-3’

ACTIN forward 5’-CGAGCACAGAGCCTCGCCTT-3’

ACTIN reverse 5’-CATCATCCATGGTGAGCTGGCGG-3’
