# Supplementary material for: The receptor tyrosine kinase AXL promotes migration and invasion in colorectal cancer
Source: PLoS One. 2017 Jul 20;12(7):e0179979. doi: 10.1371/journal.pone.0179979 (PMC5519024; doi:10.1371/journal.pone.0179979)
Supplement: S2 Table — (PDF) [file pone.0179979.s002.pdf]

GO term BP FAT		Number of genes	P value
1.	cell adhesion	152	1.9E-52
2.	biological adhesion	152	2.3E-52
3.	response to wounding	90	2.2E-22
4.	extracellular matrix organization	35	3.1E-18
5.	extracellular structure organization	42	4.2E-17
6.	vascular development	52	9.9E-17
7.	blood vessel development	50	1.0E-15
8.	cell motion	71	1.2E-14
9.	skeletal system development	52	2.6E-12
10.	inflammatory response	52	5.4E-12
11.	defense response	77	9.0E-12
12.	regulation of cell motion	38	1.3E-11
13.	regulation of cell migration	35	2.2E-11
14.	cell-cell adhesion	46	2.7E-11
15.	cell migration	46	2.7E-11

KEGG Pathway			
1.	ECM-receptor interaction	35	1.1E-20
2.	Focal adhesion	47	4.2E-16
3.	Cell adhesion molecules (CAMs)	30	4.6E-10
4.	Complement and coagulation cascades	18	3.7E-7
5.	Regulation of actin cytoskeleton	31	9.4E-6
