# Supplementary material for: The receptor tyrosine kinase AXL promotes migration and invasion in colorectal cancer
Source: PLoS One. 2017 Jul 20;12(7):e0179979. doi: 10.1371/journal.pone.0179979 (PMC5519024; doi:10.1371/journal.pone.0179979)
Supplement: S3 Table — (PDF) [file pone.0179979.s003.pdf]

GO term BP FAT		Number of genes	P value
1.	regulation of establishment of protein localization	4	1.9E-3
2.	regulation of protein localization	4	2.8E-3
3.	response to wounding	6	4.2E-3
4.	phagocytosis	3	4.4E-3
5.	phospholipid translocation	2	8.3E-3
