# Supplementary figures and images for: The receptor tyrosine kinase AXL promotes migration and invasion in colorectal cancer
Source: PLoS One. 2017 Jul 20;12(7):e0179979. doi: 10.1371/journal.pone.0179979 (PMC5519024; doi:10.1371/journal.pone.0179979)

## Bittner

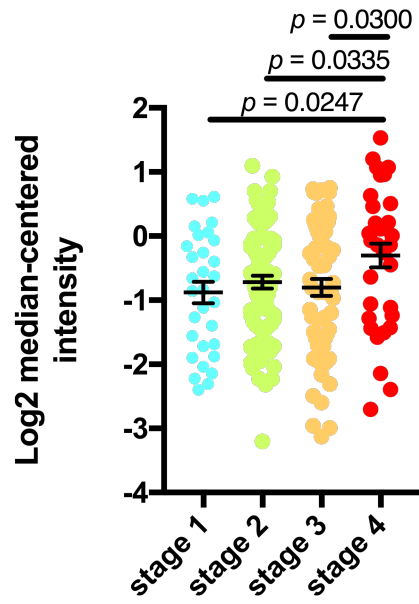

## Bittner

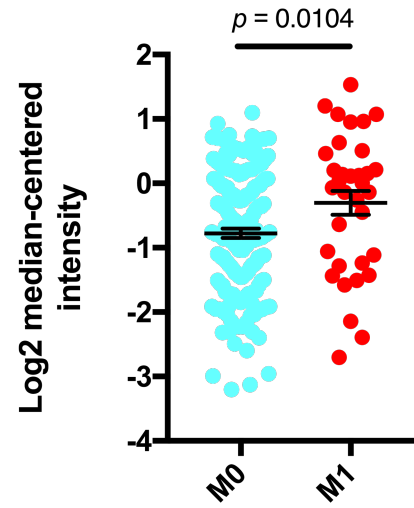

## Jorissen 3

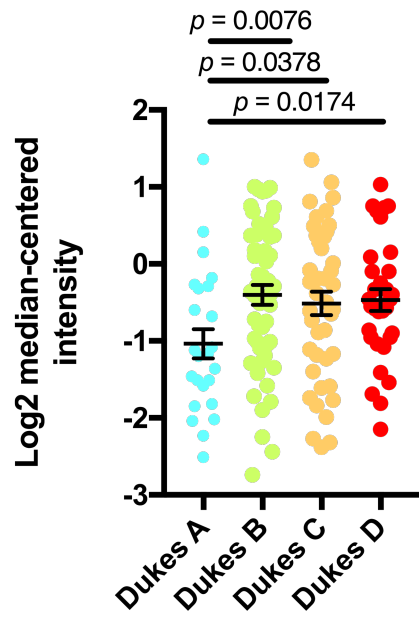

## Smith

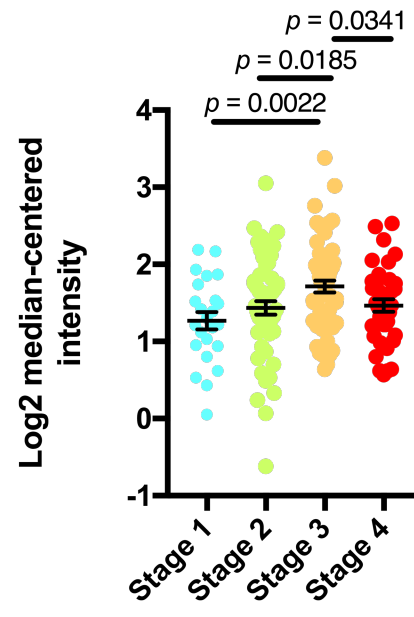

Supplement: S1 Fig — (PDF) [file pone.0179979.s004.pdf]

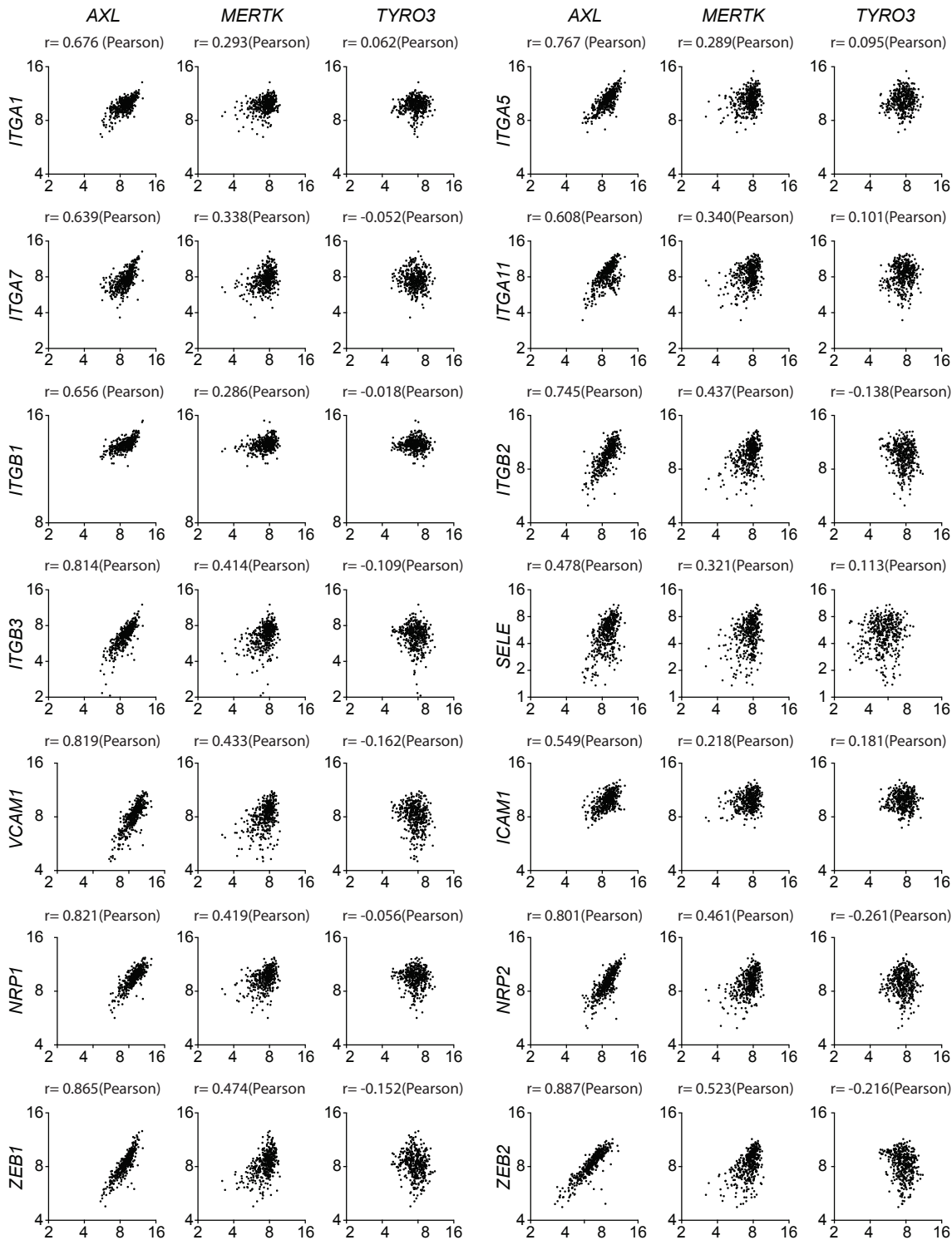

Supplement: S3 Fig — Correlation between the expression of selected cell migration-associated genes, Integrins alpha 1, 5, 7 and 11, Integrins beta 1, 2 and 3, E-selectin, VCAM1, ICAM1, Neuropilin 1 and 2 and ZEB1 and 2, and AXL, MERTK and TYRO3 in TCGA. mRNA amount is expressed as log2 RSEM. (PDF) [file pone.0179979.s006.pdf]

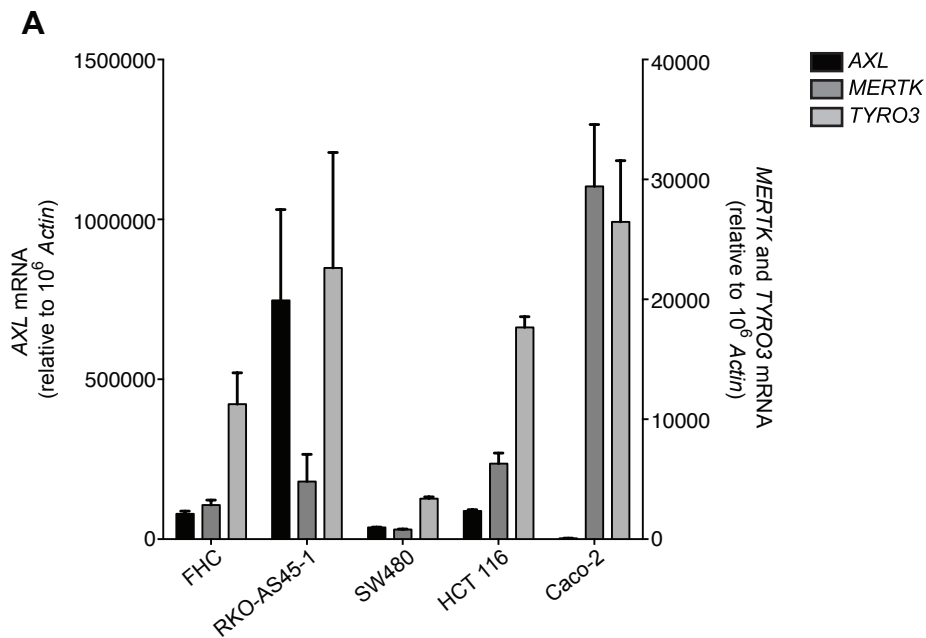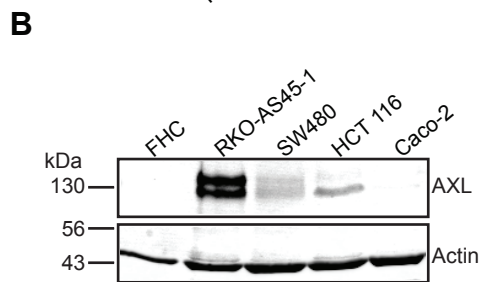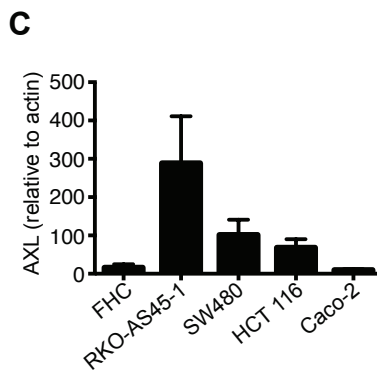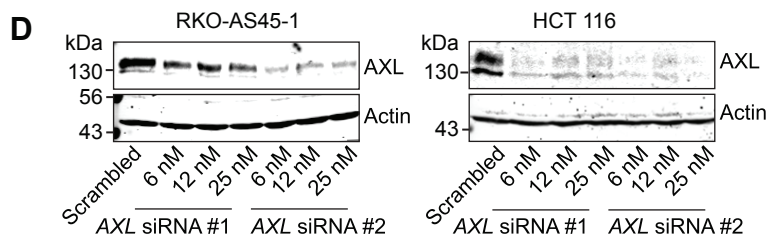

Supplement: S4 Fig — (A) expression of AXL, MERTK and TYRO3 in the indicated colon cancer cell lines, as detected by RT-qPCR. (B) representative western blot and (C) quantified AXL:ACTIN signal ratios in colon cancer cell lines. (D) representative western blot showing the efficiency of siRNA-mediated knock down of AXL in RKO-AS45-1 and HCT116 cells. Cells were transfected with scrambled or AXL siRNAs, cell lysates were collected after 48h, resolved by SDS/PAGE and immunoblotted with antibodies against AXL and ACTIN. Data are presented as individual samples or mean ± SEM of 3 independent samples. (PDF) [file pone.0179979.s007.pdf]

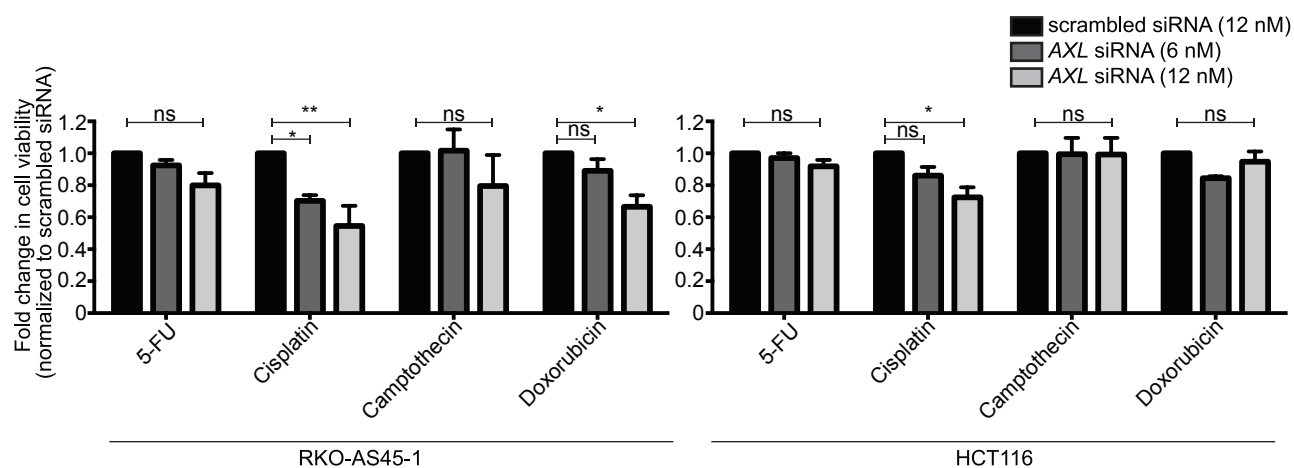

Supplement: S5 Fig — Relative change in cell viability of RKO-AS45-1 and HCT116 transfected with scrambled or AXL siRNAs and treated with LD50 concentrations of the indicated chemotherapeutic agents for 24 hours. Data is presented as representative samples or as mean ± SEM of 3 independent experiments, * p<0.05; ** p <0.01; n.s., non significant. (PDF) [file pone.0179979.s008.pdf]
